# Estimation of cause-specific mortality in Rakai, Uganda, using verbal autopsy 1999-2019

**DOI:** 10.1080/16549716.2024.2338635

**Published:** 2024-05-08

**Authors:** Dorean Nabukalu, Júlia Almeida Calazans, Milly Marston, Clara Calvert, Hadijja Nakawooya, Brendah Nansereko, Robert Sekubugu, Gertrude Nakigozi, David Serwadda, Nelson Sewankambo, Godfrey Kigozi, Ronald H Gray, Fred Nalugoda, Fredrick Makumbi, Tom Lutalo, Jim Todd

**Affiliations:** a Data management, Rakai Health Sciences Program, Rakai, Uganda; b Population Health, London School of Hygiene and Tropical Medicine, London, UK; c Centre for Demographic Studies (CED), Universitat Autònoma de Barcelona, Barcelona, Spain; d Usher Institute, University of Edinburgh, Edinburgh, UK; e Epidemiology and Biostatistics, Makerere University School of Public Health, Kampala, Uganda; f College of Health Sciences, Makerere University School of Medicine, Kampala, Uganda; g Epidemiology and International Health, Johns Hopkins Bloomberg School of Public Health, Baltimore, USA

**Keywords:** Verbal autopsy, adult mortality, Uganda, health and demographic surveillance sites, cause of death

## Abstract

**Background:**

There are scant data on the causes of adult deaths in sub-Saharan Africa. We estimated the level and trends in adult mortality, overall and by different causes, in rural Rakai, Uganda, by age, sex, and HIV status.

**Objectives:**

To estimate and analyse adult cause-specific mortality trends in Rakai, Uganda.

**Methodology:**

Mortality information by cause, age, sex, and HIV status was recorded in the Rakai Community Cohort study using verbal autopsy interviews, HIV serosurveys, and residency data. We estimated the average number of years lived in adulthood. Using demographic decomposition methods, we estimated the contribution of each cause of death to adult mortality based on the average number of years lived in adulthood.

**Results:**

Between 1999 and 2019, 63082 adults (15–60 years) were censused, with 1670 deaths registered. Of these, 1656 (99.2%) had completed cause of death data from verbal autopsy. The crude adult death rate was 5.60 (95% confidence interval (CI): 5.33–5.87) per 1000 person-years of observation (pyo). The crude death rate decreased from 11.41 (95% CI: 10.61–12.28) to 3.27 (95% CI: 2.89–3.68) per 1000 pyo between 1999–2004 and 2015–2019. The average number of years lived in adulthood increased in people living with HIV and decreased in HIV-negative individuals between 2000 and 2019. Communicable diseases, primarily HIV and Malaria, had the biggest decreases, which improved the average number of years lived by approximately extra 12 years of life in females and 6 years in males. There were increases in deaths due to non-communicable diseases and external causes, which reduced the average number of years lived in adulthood by 2.0 years and 1.5 years in females and males, respectively.

**Conclusion:**

There has been a significant decline in overall mortality from 1999 to 2019, with the greatest decline seen in people living with HIV since the availability of antiretroviral therapy in 2004. By 2020, the predominant causes of death among females were non-communicable diseases, with external causes of death dominating in males.

## Paper context

- **Main findings:** There are significant declines in mortality in people living with HIV. However, mortality in HIV-negative people increased due to non-communicable diseases in females, and injuries and external causes of death among males.
- **Added knowledge:** In this HIV-endemic area, decreasing adult mortality has been documented over the last 20 years. This paper benchmarks the changes in cause-specific mortality in this area.
- **Global health impact for policy action:** As in many African countries, more effort is needed to reduce mortality for non-communicable diseases, injuries, and external causes of death as these seem to have been neglected.

## Background

Knowledge of the numbers of deaths and understanding the causes of death is essential for documenting evidence of the demographic and epidemiological transition and planning healthcare services and interventions [1]. The available empirical evidence shows that the mortality patterns in many African countries diverge from other world regions [2]. Some studies show that many African countries are experiencing parallel burdens of communicable and non-communicable diseases (NCD), with multimorbidity becoming an important research focus [3,4]. The HIV/AIDS epidemic caused very different patterns of mortality, being the leading cause of death among adults of reproductive age in South Africa between 2000 and 2009 [5,6]. However, since the widespread use of antiretroviral therapy (ART), few studies have documented the subsequent causes of adult mortality in Africa, and adult mortality remains a neglected public health issue.

Limited data from Uganda shows a reduction in adult mortality from 132.6 per 1000 person-years in the 1990s to 7.5 per 1000 person-years in 2015, but with no estimates of cause-specific mortality [7–9]. With the scarcity of data on the cause of death in Uganda, it remains unclear where Uganda sits in the demographic transition from infectious disease burden to NCDs. This scarcity of data is partly due to the lack of adequate vital registration systems, resulting in the underreporting of vital statistics and little data on the cause of death.

The verbal autopsy (VA) tool can be used to obtain the cause of death in settings that lack adequate vital registration systems [10,11]. In such circumstances, health and demographic surveillance sites (HDSS) have a role to play in documenting deaths and collecting timely and accurate data from the VA tool [12]. VA has been widely used in HDSS in eastern [13] and southern Africa [5,6,10]. The aim of our study was to use VA in a rural population in Uganda to estimate adult mortality rates and cause-specific mortality from 2000 to 2019, stratified by age, sex, and HIV status. We used demographic decomposition methods to assess the contribution of each cause of death to adult mortality [14].

## Methods

### Study design, setting, and population

Rakai Health Sciences Program (RHSP) runs an HDSS, which is embedded in an open population-based HIV surveillance cohort (Rakai Community Cohort Study (RCCS)) in 50 communities (villages) in the greater Rakai region. The first round of data collection was a census of the whole population residing in the RCCS in 1999 [15]. This provided a framework for selection into an HIV survey for all residents aged 15–49 years [15]. All adults (18 years and above) and emancipated minors (children aged 15–17 years living independently of parents or guardians) were asked for written informed consent. Unemancipated minors (children aged 15–17 years living in households under the care of parents or guardians) were asked for assent, and their parents/guardians were asked for informed written consent on their behalf. Consent is obtained to participate in the census and survey activities. Participants give further consent to conduct HIV testing. All consenting participants were tested for HIV, and those positive were offered counselling and treatment [15]. HIV sero status was detected using two enzyme immunoassays, with western blot and/or polymerase chain reaction confirmation. Subsequent rounds of data collection in the RCCS involved the census and survey and were repeated at approximately 18-month intervals. At census, information collected includes births, social economic status, deaths, migrations, and pregnancy outcomes. At survey, information collected is about HIV risky behaviour, social behavioural characteristics, pregnancy status, and HIV status. The design and conduct of the RCCS have been described elsewhere [15].

As of 2019, the RCCS covered approximately a population of 98,000, of which 60% are aged 15 years and above. The percentage of females in the population is 51%. HIV prevalence in the periods before and after ART establishment was as follows: i) preceding ART services (1999–2004)−13.1%, and three periods following ART availability, i.e. ii) 2005–2009–15.7%, iii) 2010–2014–16.9%, and iv) 2015–2019–18.7%. In response to this high HIV prevalence, the Rakai Health Sciences Program (RHSP), with support from the President’s Emergency Plan for AIDS Relief (PEPFAR), has provided a comprehensive Antiretroviral Therapy (ART) program in the district since 2004. The effectiveness of this program in adults is undisputed [7,16].

This analysis included 30 communities that were consistently followed from 1999 to 2019. Information from the household census was used to update household members’ residency and deaths. It is important to note that in this cohort, HIV testing is done for individuals aged 15–49 years, and the most recent HIV status to the time of death is reported.

### Data collection and management

Participants aged 15–60 years from the household census were included in this study. HIV survey participants (15–49 years) provided a venous blood sample, while HIV status for those aged 50 years and above relied on the most recent tests done before age 50. Information on signs and symptoms prior to death was collected by verbal autopsy (VA) at least six weeks after a death had occurred. Two different VA instruments were used: From 1999 to 2013, the site used a customized VA tool administered by trained interviewers. This tool was developed by the Rakai Health Sciences Program (research department) and had questions relating to the signs and symptoms prior to death. From 2014 onwards, the site used the standard World Health Organization (WHO) VA questionnaire with one of two trained clinicians collecting the information about the deceased. To assign a cause of death from the VA, two independent physicians reviewed questionnaires and used the ICD-10 codes [17] for disease classification of the underlying cause of death [18]. Where there was a disagreement between the two, a third physician would sit with the two, review the VA, and agree on a cause of death. Failure to assign a cause of death from this meeting results in an indeterminate cause of death being assigned. The assigned causes of death were classified into WHO broad underlying causes of death groups: I-Communicable diseases and maternal conditions, II-Non-Communicable diseases, III-Injuries, and IV-Other causes of death. The Group I were further subclassified into i) HIV/AIDS/TB, ii) maternal causes and iii) other communicable diseases [19]. For this analysis, the study period was categorized into four sub-periods: preceding ART services (1999–2004) and three periods following ART introduction (specifically, 2005–2009, 2010–2014, and 2015–2019).

### Statistical analysis

We allocated person-time to HIV infection by the time from the last negative to the first positive HIV test. The time following a positive HIV was estimated until censoring or death. The time following the last negative test is considered negative for five years, after which it is classified as unknown. The time between two HIV-negative tests is counted as negative, no matter how long the interval between tests.

The life table probabilities of dying between the ages 15 and 60 (45q15) stratified by sex were computed and plotted by age (5-year age groups) and calendar years. Survival analysis was used to estimate crude mortality rates with 95% confidence intervals (CI) by sex, age, and HIV status.

Deaths per causal category, age, and sex were obtained from physician reviews and linked to HIV surveys (using unique study identifiers) to obtain HIV status at death. The cause-specific mortality fractions (CSMF) were estimated as the number of deaths due to a specific cause divided by the total number of deaths with a VA, expressed as a proportion and calculated by age, sex, and HIV status. The CSMF was then applied to the stratified age-specific mortality rates to estimate stratified cause-specific mortality rates. Percentile-based confidence intervals for the CSMF were estimated by bootstrapping with 1,000 replications.

We used decomposition analysis techniques proposed by Home [14] to analyse the average number of years lived in adulthood (ANYLA) to measure adult mortality. The ANYLA was estimated as the ratio between two life table functions, and it can be interpreted as the average number of years a 15-year-old is expected to live up to age 60 (_45_e_15_) under the prevailing mortality conditions. It is given by the formula:

$${\;}_{45}e_{15}{=}_{45}L_{15}/l_{15}.$$

Where _45_L_15_ represents the total number of person-years lived between the ages 15 and 60, and l_15_ represents the number of people living at age 15. The ANYLA was computed and plotted by sex, calendar year, and HIV status. The variation in ANYLA was decomposed to establish the contribution of each cause of death to changes in adult mortality using a model proposed by Horiuchi [20] and has been adopted in previous studies [21–23]. Theoretically, in decomposition analysis, a change in the dependent variable (change in ANYLA) can be expressed as a sum of the effects of the covariates (cause-specific mortality rates). For this decomposition analysis, the ANYLA was compared between 2000 (the first full year of data collection) and 2019. All analyses were done in Stata V15 (College Station, Texas) and R version 4.3.0.

## Results

**Figure 1.**
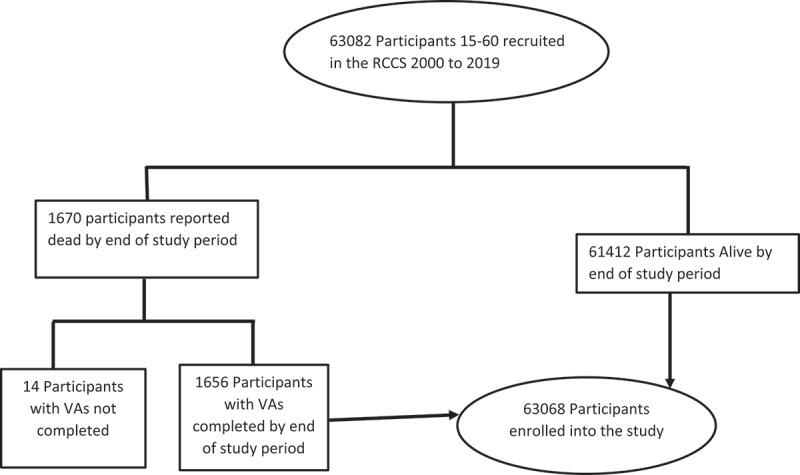
RCCS cohort composition for the cause of death analysis.

Source: Rakai Community Cohort Study, 1999 to 2019

Between 1999 and 2019 inclusive, a cumulative number of 63,082 adults (15–60 years) resided in the demographic surveillance area. Of these, 14 were excluded from this study as they had died but did not have a VA, leaving a total of 63,068 adults (Figure 1). Of those included, they jointly contributed 295,137 person-years of observation (pyo) time and 1656 deaths to the analyses giving an overall crude death rate of 5.60 (95% CI:5.33–5.87) per 1000 pyo. Table 1 shows a breakdown of the crude death rates for each analysis period.

**Table 1.** Characteristics of the study population, and death rates (per 1000 pyo) at different periods (RCCS) - Rakai Community, Uganda (1999 to 2019).

	Individuals	Person-Years	Deaths	1999–2004 Rate (95%-CI)	2005–2009 Rate (95%-CI)	2010–2014 Rate (95%-CI)	2015–2019 Rate (95%-CI)
All		295137	1656	11.41(10.61–12.28)	5.55(5.01–6.13)	3.58(3.19–4.01)	3.27(2.89–3.68)
**Males**							
15–24	17173	55690	93	2.65(1.89–3.71)	1.71(1.13–2.60)	1.11(0.69–1.79)	1.35(0.87–2.09)
25–34	7475	39850	268	11.60(9.59–14.03)	7.39(5.88–9.31)	4.43(3.35–5.87)	4.08(2.99–5.57)
35–44	3134	28468	283	23.72(19.78–28.46)	10.75(8.46–13.66)	6.59(5.09–8.55)	4.94(3.66–6.66)
45–49	749	8483	94	23.53(16.54–33.45)	12.97(8.46–1989)	8.78(5.78–13.34)	6.58(4.24–10.19)
50–59	642	8389	126	27.52(19.67–38.52)	12.93(8.59–19.46)	10.79(7.34–15.84)	14.50(10.76–19.55)
All males	29173	140880	864	10.92(9.79–12.18)	6.37(5.56–7.30)	4.29(3.69–4.98)	4.22(3.63–4.92)
**Females**							
15–24	22441	62408	130	3.52(2.68–4.62)	2.59(1.88–3.56)	1.54(1.05–2.26)	0.87(0.52–1.48)
25–34	7453	44379	323	20.63(17.89–23.81)	6.21(4.91–7.87)	3.09(2.27–4.22)	2.31(1.58–3.39)
35–44	2561	26934	196	19.91(16.35–24.25)	7.84(5.77–10.65)	3.52(2.42–5.14)	3.19(2.22–4.59)
45–49	604	8311	49	14.94(10.09–22.11)	3.41(1.62–7.15)	3.75(1.87–7.49)	3.68(1.91–7.06)
50–59	836	12224	94	11.06(7.73–15.82)	6.16(3.83–9.91)	7.33(4.95–10.85)	6.58(4.33–9.99)
All females	33895	154257	792	11.85(10.74–13.08)	4.80(4.14–5.58)	2.93(2.46–3.48)	2.37(1.95–2.89)
**HIV status**							
**15–59**							
Negative	19693	118535	290	2.44(1.89–3.14)	2.72(2.17–3.41)	2.12(1.68–2.67)	2.56(2.06–3.17)
Positive	2935	15459	384	61.07(53.47–69.77)	23.52(18.92–29.25)	11.86(9.01–15.59)	8.41(6.04–11.71)
Unknown	40440	161142	982	12.70(11.56–13.95)	5.97(5.24–6.81)	3.89(3.36–4.52)	3.30(2.81–3.88)

Source: Rakai Community Cohort Study, 1999 to 2019.

Table 1 shows that women contributed 52% of the person-years of exposure to the study, and the HIV status is known for around 46% of the total person-years lived. The overall crude death rate decreased from 11.41 (95% CI: 10.61–12.28) to 3.27 (95% CI: 2.89–3.68) per 1000 pyo between 1999–2004 and 2015–2019, respectively. Great declines in mortality were observed in people living with HIV (aged 15–59 years), from a high of 61.07 per 1000 pyo (95% CI: 53.47–69.77) in 1999–2004 to a low of 8.41 per 1000 pyo (95% CI: 6.04–11.71) in 2015–2019.

**Figure 2.**
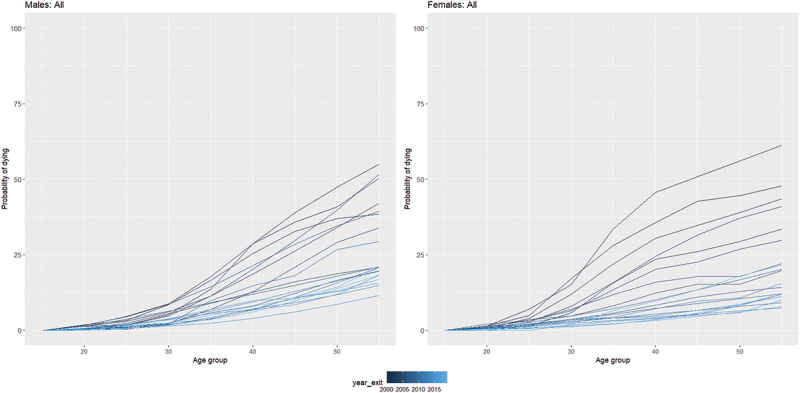
Mortality rates: Age-specific probability of dying between age 15 and 60 - Rakai Community, Uganda (1999 to 2019).

*Source: Rakai Community Cohort Study, 1999 to 2019*

Figure 2 shows changes in the age-specific probability of dying between age 15 and 60 by sex over the study period. The probability of dying decreased substantially over the years (higher probability with darker blue than lighter blue), and these declines have been more dramatic in women than men.

Cause-specific mortality rates obtained from the VA causes of death for each period are displayed in Table 2. HIV/AIDS/TB had the highest mortality rate for both males and females except in the period 2015–2019. Among female deaths between 2015 and 2019, non-communicable causes of death had the highest mortality rate (0.78 per 1000 pyo), followed by other causes of death (0.55 per 1000 pyo). In contrast, male deaths over the same time period were primarily due to other causes of death (1.14 per 1000 pyo) followed by external causes of death (1.07 per 1000 pyo) (Table 2). A similar pattern is also observed with the cause-specific mortality fractions, as displayed in Table S1. In addition, Figure S2 shows the top seven causes of death in each broad death category. Neoplasms were the leading cause of death among non-communicable diseases, while road traffic accidents were the leading cause of external deaths.

**Table 2.** Cause-Specific Mortality Rates (per 1000 pyo) in the RCCS – Rakai Community, Uganda (1999 to 2019).

Years	HIV/AIDS/TB	Other Communicable	Non Communicable	External causes	Other causes	Undetermined	
**Males**							
1999–2004	3.81	3.44	1.43	0.78	0.14	1.33	
2005–2009	2.16	1.64	0.96	1.08	0.15	0.37	
2010–2014	1.43	0.73	0.93	0.7	0.3	0.2	
2015–2019	0.81	0.18	0.92	1.07	1.14	0.1	
	HIV/AIDS/TB	Other Communicable	Non Communicable	External causes	Other causes	Maternal causes	Undetermined
**Females**							
1999–2004	4.72	4	0.9	0.18	0.21	0.3	1.53
2005–2009	1.51	1.48	0.61	0.2	0.08	0.31	0.61
2010–2014	1	0.51	0.47	0.23	0.21	0.37	0.14
2015–2019	0.43	0.19	0.78	0.12	0.55	0.26	0.05

Between 2000 and 2019, overall, the average number of years lived in adulthood (ANYLA) increased by 6.4 years in males and 6.7 years in females. A stratification by HIV status showed that women living with HIV gained 22 years (17.4 to 39.4 years) compared to men living with HIV who gained 5 years only (28.5 to 33.5 years) (Table 3). Conversely, between 2000 and 2019, ANYLA slightly reduced from 39.8 to 39.3 years for HIV-negative females (−0.5 years) and from 38.7 to 37.9 years for HIV-negative males (−0.9 years). Figure S1 shows trends in ANYLA, stratified by sex and HIV Status. Consistently, ANYLA remained higher in participants who were HIV negative compared to those who were HIV positive or had unknown HIV status at the time of death. In Figure S1, ANYLA for participants living with HIV (both females and males) first decreased in the years earlier than 2004 and, after that, increased consistently to a high of 40 years in 2019.

**Table 3.** Average number of years lived in adulthood (RCCS) - Rakai Community, Uganda (1999 to 2019).

	Male				Female			
Period	Whole Population	Positive	Negative	Unknown	Whole Population	Positive	Negative	Unknown
2000	31.5	28.5	38.8	29.7	31	17.4	39.8	29.5
2019	37.9	33.5	37.9	38.9	37.7	39.4	39.3	39.3
diff	6.4	5	−0.9	9.2	6.7	22	−0.5	9.8

Source: Rakai Community Cohort Study, 1999 to 2019.

Figure 3 shows an age-cause decomposition of the average number of years lived in adulthood between 2000 and 2019 by sex and HIV status. Overall, declines in cause-specific mortality of HIV/AIDS/TB and other communicable diseases explained a large proportion of the increase in the ANYLA between 2000 and 2019 for both males and females (Figure 3(a)). Non-communicable diseases were responsible for a reduction in the ANYLA in all females. At the same time, external and other causes of death were responsible for the reduction in ANYLA in all males. The increase in ANYLA for people living with HIV is largely due to a reduction in HIV/AIDS/TB mortality plus other communicable diseases (Figure 3(b)). While this is true for women living with HIV aged 25 years and above, we observe a reduction in ANYLA because of a slight increase in mortality for men living with HIV caused by external causes of death and other causes.

**Figure 3.**
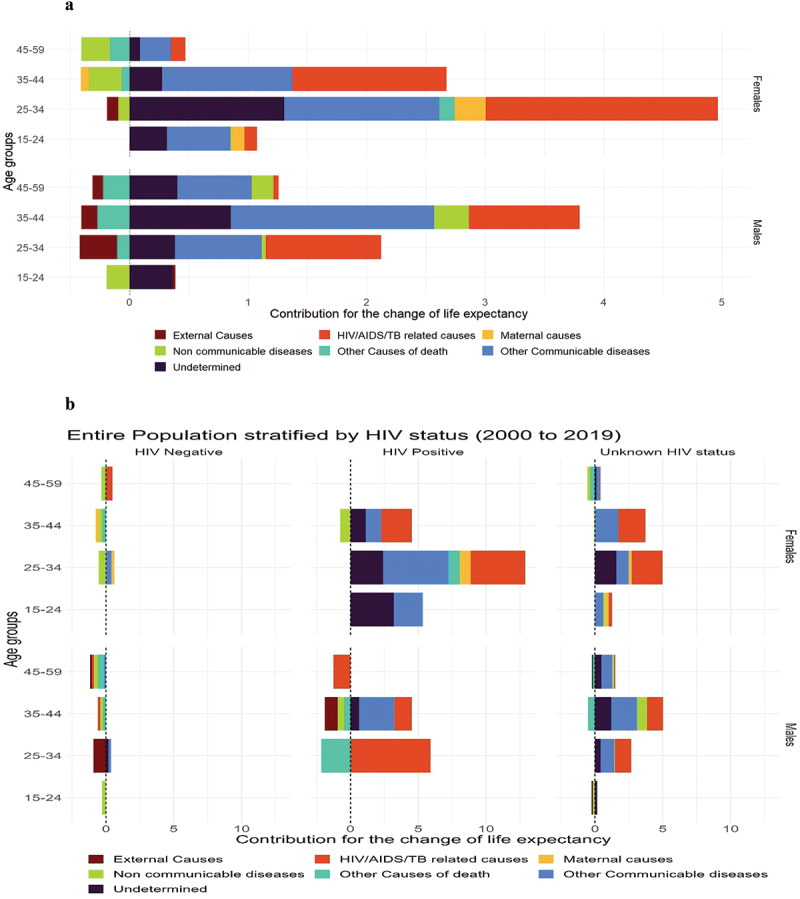
Decomposition of average number of years lived in adulthood, in the entire population (figure 3a) and stratified by HIV status (figure 3b) and by ART establishment periods (figure 3c and figure 3d) - Rakai Community, Uganda (1999 to 2019).

Source: Rakai Community Cohort Study, 1999 to 2019

Further, the reduction in ANYLA is due to an increase in cause-specific mortality in HIV-negative individuals fuelled by NCDs (at older ages in females and younger ages in males), external causes (25–34 years in both females and males), and other causes of death (for females aged 35–44 and males aged 45–59 years) (Figure 3(b)). Figure 3(c) shows an age-cause decomposition of ANYLA in the pre-ART period (2000 to 2004). In females, we observe large reductions in ANYLA due to high cause-specific mortality rates of mainly HIV/AIDS/TB-related causes and NCDs. In males, high cause-specific mortality rates of almost all conditions were responsible for reductions in ANYLA, especially in HIV-negative and HIV-positive individuals. In the era after ART establishment (2005–2019), large increases in ANYLA were observed as a result of lower cause-specific mortality rates of almost all conditions except NCDs in females aged 25 years and above for both HIV-negative women and women living with HIV (Figure 3(d)). In males, reductions in ANYLA are explained by high mortality rates of other causes of death in people living with HIV and mainly external causes of death in HIV-negatives (Figure 3(d)).

**Figure 3.**
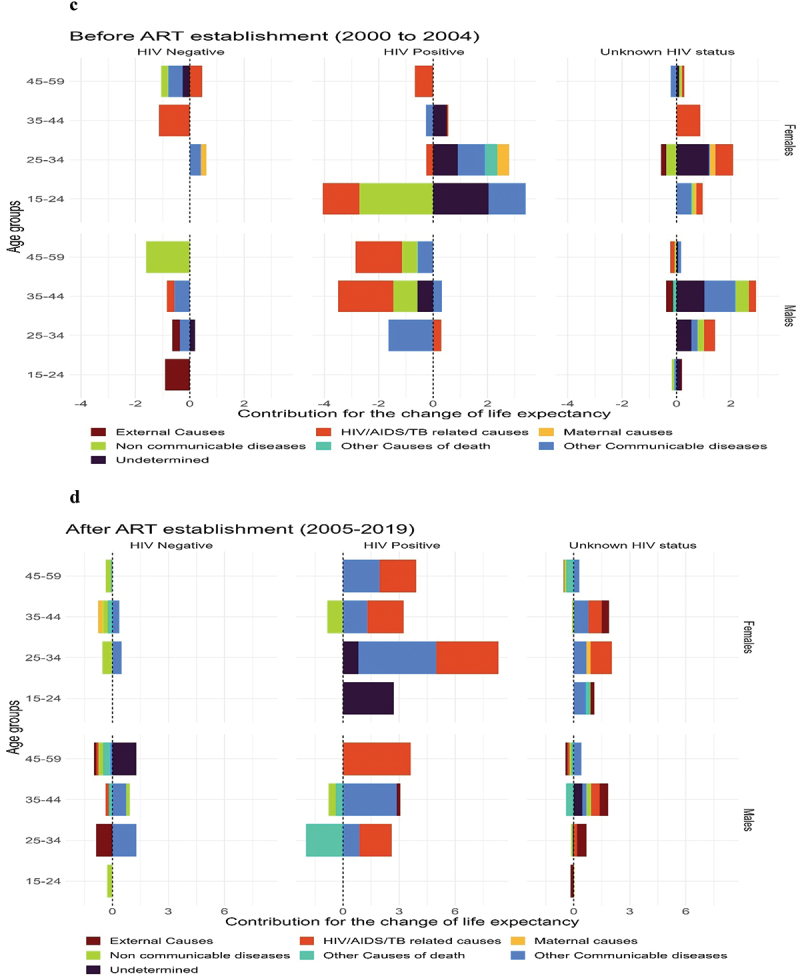
(continued).

## Discussion

Between 1999 and 2019, heterogeneity was observed in adult mortality by sex and HIV status. Large reductions in mortality have been observed in people living with HIV, particularly those aged 25 to 44 years, an age range where HIV-associated mortality was common [5,6]. This decline is due to increased survival within this cohort, and findings are similar to other studies conducted earlier in the region and elsewhere [7,24,25], implying a positive impact of the ART program on adult mortality. Greater declines in mortality in people living with HIV have been observed in females than in males. This is partly because women are known to have higher rates of HIV testing and counselling and, higher ART coverage rates, earlier treatment initiation, and lower attrition and mortality rates on ART [26–29].

On the other hand, the mortality of HIV-negative individuals is increasing, as observed in the reduction of the average number of years lived in adulthood (ANYLA) in this group. This pattern observed in adult mortality can be explained by the different causes of death, like NCDs and external causes of death, as illustrated in the results. Other studies around the region observed a similar pattern in cause-specific adult mortality, though no significant changes in the overall mortality rates of the HIV-negative individuals were observed.

Results have further shown how treating several diseases has increased ANYLA in this cohort. The biggest improvement appears to be in men with unknown HIV status (but not in women with unknown HIV status). This is because increases in the number of years lived were most likely to occur in those aged 50–60. In people living with HIV, treatment of HIV/AIDS/TB and other communicable diseases has had the biggest contribution to the ANYLA in both females and males, thus agreeing with other studies that have documented the positive impact of the ART program in this region and sub-Saharan Africa at large [7,24,25,30–33].

In HIV-negative males, external causes of death are responsible for the decline in the average number of years lived in adulthood, mostly in adolescents and young adult males 34 years and below. In Uganda, motorcycle transportation (– locally known as boda–boda riding) is a booming business, mainly operated by young people, and our results have shown road traffic accidents as the leading causes of death in this category. Therefore, the considerable contribution of injuries in this age group could be associated with the increase in motorist accidents in the country, where victims can either be riders or passengers or both [34,35].

With these results, we can observe that in this cohort, the epidemiological transition from infectious disease mortality to the rise in non-communicable disease mortality is beginning and needs to be studied further, especially in population-based studies. Changes in disease burden are already observable in most African countries, and the global prevalence of NCDs has risen over the past decade, especially in low- and middle-income countries (including Uganda). If this is not controlled, they are predicted to become the leading cause of death in Africa [36]. In this study, NCDs (with neoplasms taking the lead) are observed to be responsible for the reduction in adult years lived, especially in females. This may be linked to the country’s inadequate management of NCDs due to limited resources, as highlighted by a recent study conducted in the country [37]. The Uganda Ministry of Health prioritizes NCD prevention, early diagnosis, and management. However, health facilities have limited capacity to manage and implement interventions targeting the growing NCD burden [38]. Thus, emphasizing the need for interventional programs and systems to educate better, characterize, and care for NCDs.

A fundamental limitation of this study is the use of verbal autopsy for assigning the cause of death, given that it is a relatively imprecise tool for obtaining the cause of death, particularly in settings where there are low levels of diagnostic tests. Given these limitations, we have used relatively broad cause of death categories rather than more specific diagnoses. The proportion of confirmed HIV status in the study population was only 46% because HIV status was only actively updated for those aged 15–49 years. As such, anyone who seroconverted after 49 years old ended up being classified as having unknown HIV status, thus underestimating the impact of the HIV/AIDS treatment and prevention programs in the area. There are also a number of strengths to this work. The main strength of this work is that in a population cohort, deaths that occurred outside the health facility could be easily tracked compared to a health facility study, thus minimizing the underestimates of the rates that could occur if only health facility-based deaths were considered. There was also excellent VA coverage, with almost all deaths having a VA, which gives an almost complete picture of what was happening in the region.

## Conclusion

This study highlights the changes in adult mortality by causes of death in a population cohort and how these changes might impact the average number of years lived in adulthood. This cohort has great heterogeneity in adult mortality levels across sex and HIV status. Overall, significant declines in mortality have been observed and are largely driven by declines in HIV-related mortality. Mortality in HIV-negative people is greatly affected by conditions that seem to have been neglected over time, with NCDs being the predominant cause of death in females and among males, injuries and external causes of death dominate, thus evidence of an early transition (increase in NCDs and external/other causes). If these causes of death are left unattended for a long time, it might change the disease burden in this region. This study presents a benchmark of cause-specific mortality in this region; thus, future studies are needed to continue monitoring changes in mortality patterns, adjust health policy, and plan to meet the community’s changing health needs.

## Supplementary Material

SUPPLEMENTARY MATERIAL_paper1.docx
