# Supplementary material for: Estimation of cause-specific mortality in Rakai, Uganda, using verbal autopsy 1999-2019
Source: Glob Health Action. 2024 May 8;17(1):2338635. doi: 10.1080/16549716.2024.2338635 (PMC11080674; doi:10.1080/16549716.2024.2338635)
Supplement: SUPPLEMENTARY MATERIAL_paper1.docx [file ZGHA_A_2338635_SM0614.docx]

# SUPPLEMENTARY MATERIAL

**Figure S1: Average number of years lived between 15 and 60 years for males and females by HIV_status**


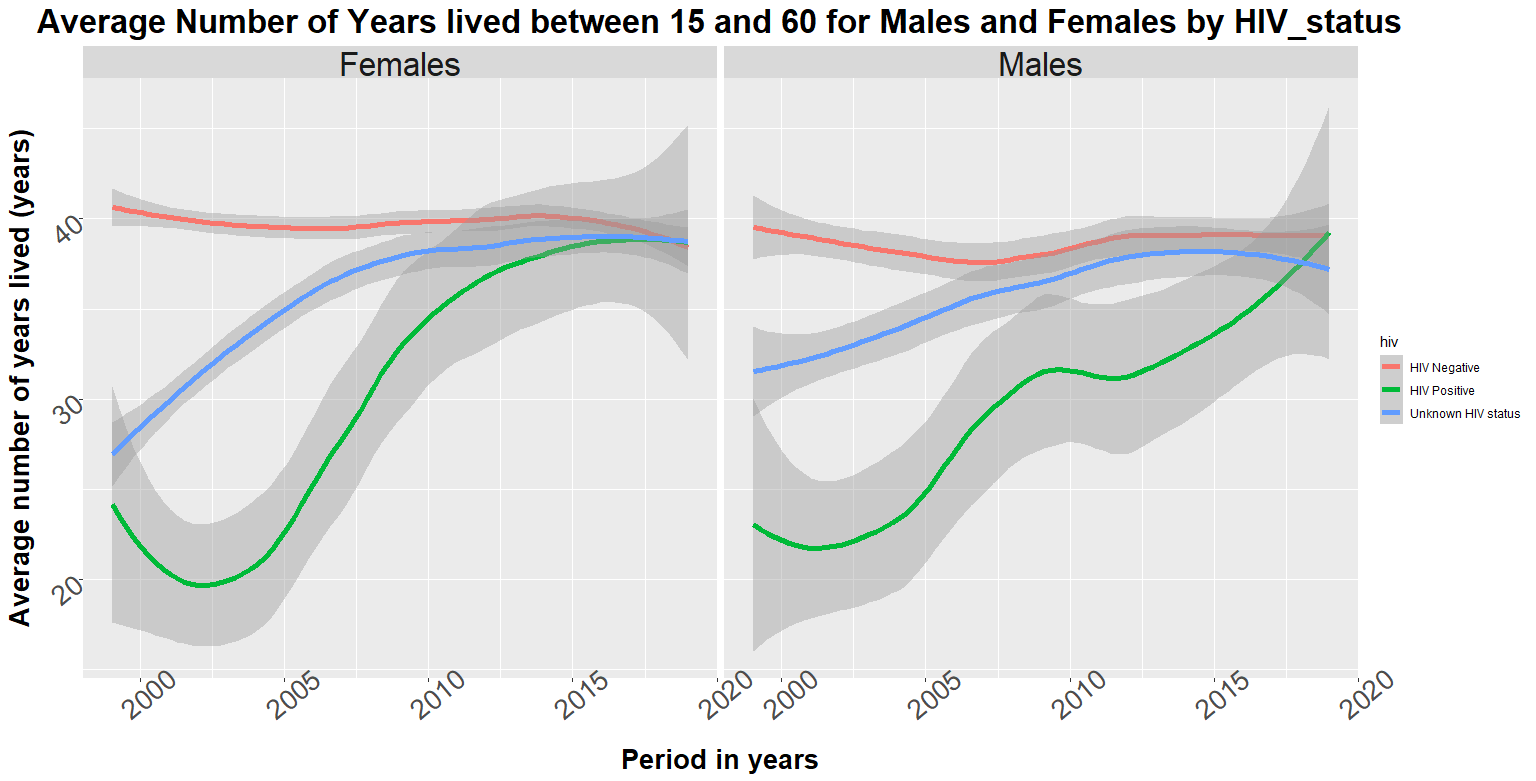


**Figure S2: Top seven causes of death for each broad category**

**
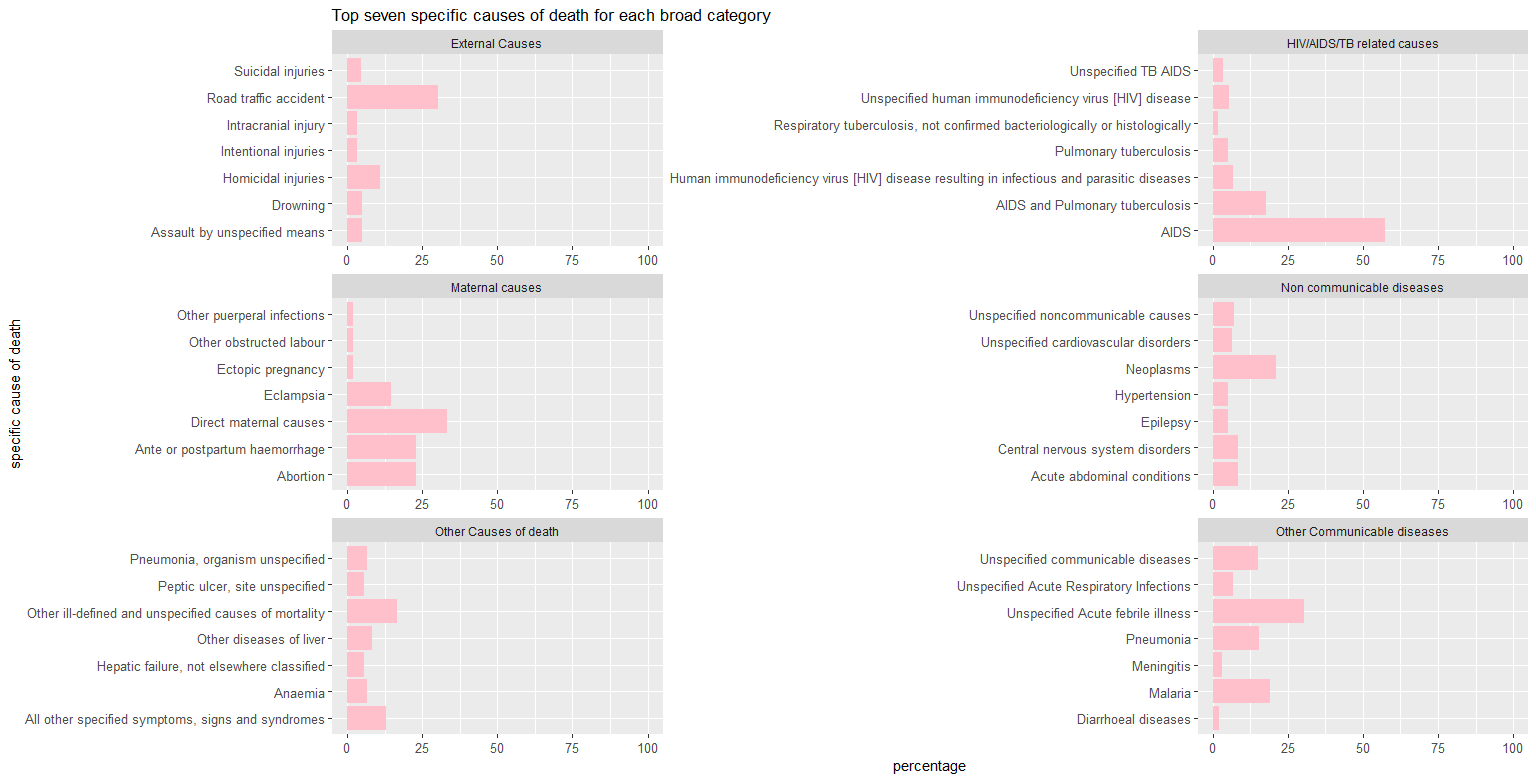
**

Injuries = Injuries and External causes of death

Others = Other causes of death

**Table S1 Cause specific Mortality Fractions in the RCCS 1999 to 2019**

| **Females** | | | | | | | |
| --- | --- | --- | --- | --- | --- | --- | --- |
| **Years** | **HIV/AIDS/TB** | **Other Communicable** | **Non Communicable** | **External causes** | **Other causes** | **Maternal causes** | **Undetermined** |
| 1999-2004 | 0.4 | 0.34 | 0.08 | 0.02 | 0.02 | 0.03 | 0.13 |
| 2005-2009 | 0.31 | 0.31 | 0.13 | 0.04 | 0.02 | 0.06 | 0.13 |
| 2010-2014 | 0.34 | 0.17 | 0.16 | 0.08 | 0.07 | 0.13 | 0.05 |
| 2015-2019 | 0.18 | 0.08 | 0.33 | 0.05 | 0.23 | 0.11 | 0.02 |
| **Males** | | | | | | | |
|  | **HIV/AIDS/TB** | **Other Communicable** | **Non Communicable** | **External causes** | **Other causes** | **Undetermined** | |
| 1999-2004 | 0.35 | 0.31 | 0.13 | 0.07 | 0.01 | 0.12 |  |
| 2005-2009 | 0.34 | 0.26 | 0.15 | 0.17 | 0.02 | 0.06 |  |
| 2010-2014 | 0.33 | 0.17 | 0.22 | 0.16 | 0.07 | 0.05 |  |
| 2015-2019 | 0.19 | 0.04 | 0.22 | 0.25 | 0.27 | 0.02 |  |

**Table S2: Cause of death (CoD) classification (RCCS 1999-2019)**

| **CoD groups in thesis** | **ICD-10 code** |
| --- | --- |
| HIV/AIDS/TB | B20-B24; A15-A16 |
| Non-Communicable diseases | D55-D89; E00-E07; E10-E35; E50-E90; F00-F99; G06—G37; G40-G41; G50- G99; H00-H95; J30-J99; K00-K31; K35-K38; K40-K93; L00-L99; M00-M99; N00-N99; R00-R94; C00—C26; C30-C58; C60-D48; D57; I00-I15; I20-I52; I60- I99 |
| Other communicable diseases | A00-A09; A17-A99; B00-B19; B25-B99; D50-D64; E40-E46; G00-G05; J00-J22 |
| Injuries and External causes | S00-T99; V01-V99; W00-W99; X00-X99; Y00-Y98 |
| Other causes | R00-R99; O00-O08; O10-O16; O20-O99 |
